# A β-Alanine Catabolism Pathway Containing a Highly Promiscuous ω-Transaminase in the 12-Aminododecanate-Degrading Pseudomonas sp. Strain AAC

**DOI:** 10.1128/AEM.00665-16

**Published:** 2016-06-13

**Authors:** Matthew Wilding, Thomas S. Peat, Janet Newman, Colin Scott

**Affiliations:** a CSIRO Land and Water, Black Mountain, Canberra, Australian Capital Territory, Australia; b CSIRO Food and Nutrition, Black Mountain, Canberra, Australian Capital Territory, Australia; c CSIRO Manufacturing, Parkville, Victoria, Australia; University of California—Davis

## Abstract

We previously isolated the transaminase KES23458 from Pseudomonas sp. strain AAC as a promising biocatalyst for the production of 12-aminododecanoic acid, a constituent building block of nylon-12. Here, we report the subsequent characterization of this transaminase. It exhibits activity with a broad substrate range which includes α-, β-, and ω-amino acids, as well as α,ω-diamines and a number of other industrially relevant compounds. It is therefore a prospective candidate for the biosynthesis of a range of polyamide monomers. The crystal structure of KES23458 revealed that the protein forms a dimer containing a large active site pocket and unusual phosphorylated histidine residues. To infer the physiological role of the transaminase, we expressed, purified, and characterized a dehydrogenase from the same operon, KES23460. Unlike the transaminase, the dehydrogenase was shown to be quite selective, catalyzing the oxidation of malonic acid semialdehyde, formed from β-alanine transamination via KES23458. In keeping with previous reports, the dehydrogenase was shown to catalyze both a coenzyme A (CoA)-dependent reaction to form acetyl-CoA and a significantly slower CoA-independent reaction to form acetate. These findings support the original functional assignment of KES23458 as a β-alanine transaminase. However, a seemingly well-adapted active site and promiscuity toward unnatural compounds, such as 12-aminododecanoic acid, suggest that this enzyme could perform multiple functions for Pseudomonas sp. strain AAC.

**IMPORTANCE** We describe the characterization of an industrially relevant transaminase able to metabolize 12-aminododecanoic acid, a constituent building block of the widely used polymer nylon-12, and we report the biochemical and structural characterization of the transaminase protein. A physiological role for this highly promiscuous enzyme is proposed based on the characterization of a related gene from the host organism. Molecular dynamics simulations were carried out to compare the conformational changes in the transaminase protein to better understand the determinants of specificity in the protein. This study makes a substantial contribution that is of interest to the broad biotechnology and enzymology communities, providing insights into the catalytic activity of an industrially relevant biocatalyst as well as the biological function of this operon.

## INTRODUCTION

Enzymes are typically thought of as highly selective, making it possible to avoid side reactions associated with analogous (nonenzymatic) chemical reactions and produce high yields of single products. However, it is perhaps underappreciated that enzymes can possess very broad substrate specificities, a key requirement for their evolutionary adaptability, as this allows for the development of new catalytic capabilities when required for survival or competitive advantage (1–3). For example, the ability of microorganisms to degrade nonnatural chemicals, such as pesticides or synthetic antibiotics, has resulted in new metabolic pathways for resistance and catabolism over relatively short periods of evolutionary time (4, 5). Although these proteins often evolve for catabolic purposes in the microorganisms involved, these newly evolved functions represent an opportunity from a biocatalytic perspective.

ω-Transaminases (ω-TAs) are renowned for their promiscuity and have exhibited catalytic prowess in the synthesis of unnatural compounds (6). The ability of these enzymes to act on foreign molecules has drawn widespread interest from both academia and industry, and while they are used widely in the synthesis of chiral building blocks (7), they can also be used to produce more complex synthetic pharmaceuticals. For example, sitagliptin, an antidiabetes compound, can be synthesized using a transaminase-mediated process which provides improvements in both yield, enantiomeric excess, and productivity relative to alternative chemocatalytic methods (8). Transaminases, including ω-TAs, are pyridoxal-5′-phosphate (PLP)-dependent enzymes that transfer amine groups from molecules containing a primary amine (the amine donor) to acceptor molecules, aldehydes or ketones (9). PLP acts as a carrier for the amine group, forming pyridoxamine-5′-phosphate (PMP) as an intermediate in the reaction. The ability to install amine groups, often in an enantioselective manner, is highly attractive, and when used to synthesize expensive products using relatively cheap amine donors such as isopropylamine, these methods can also become more economical. Although ω-transaminases are reported to have some limitations, such as low thermotolerance, substrate and product inhibition, and equilibrium-controlled yields, complementary methods have been developed to address these issues (7, 10–12). In addition, there are a number of X-ray structures available for ω-TAs, and despite having highly conserved tertiary and quaternary structures, the enzymes exhibit conformational changes upon binding, and this conformational heterogeneity has been reported to make significant contributions to substrate specificity (13). Given the low homology (<30%) among the class (14, 15), an understanding of the sequence-function relationship of these enzymes can be challenging and is typically only possible with a detailed understanding of the interactions between an enzyme and its substrate (8, 16).

Previously, we characterized a number of transaminases from Pseudomonas sp. strain AAC (NCBI accession number PRJNA248604), some of which were able to metabolize 12-aminododecanoic acid, a nonnatural compound with potential as a building block for industrial nylon-12 synthesis (17). Hazards associated with the synthesis of nylon-12 (18), as well as global shortages of the material in recent years (19), have afforded opportunities for the application of biocatalysis within the materials field (20), and a number of examples have been reported to date (21, 22). However, while most organisms are unable to metabolize 12-aminododecanoic acid, the Pseudomonas sp. strain AAC bacterium contains three enzymes with activity against the compound. Catalytic promiscuity is often thought to be the result of an enzymatic activity that was acquired coincidentally and evolved under selective pressure. In this case, the substrate is neither toxic nor abundant in the environment, and it is unlikely that there was a strong selective pressure to evolve with a metabolic activity against the compound. Examples of secondary reactions which provide no apparent benefit and are only encountered under laboratory conditions, such as the dechlorination of chloropurines by adenosine deaminase (23), have been reported, and without further evidence it is more likely that this is the case for Pseudomonas sp. strain AAC. However, the discovery of three genes encoding proteins with this activity within the same organism suggests that the compound has a broader metabolic context, or that the genes coevolved from a single parent gene (24).

As stated above, among Pseudomonas sp. strain AAC proteins, three transaminases were identified with 12-aminododecanoic acid activity: KES24511, KES23458, and KES23360. The most promising candidate biocatalyst in terms of ease of protein production, solubility, and activity was KES23458, and here we report the subsequent characterization of that protein.

## MATERIALS AND METHODS

### Materials.

A codon-optimized synthetic gene encoding KES23460 was synthesized by Life Technologies (Germany). All chemicals were purchased from Sigma-Aldrich Co. (USA).

### Cloning, expression, and purification.

Cloning of KES23458 from genomic DNA has been described previously (17). Protein overexpression was achieved by growing a culture of Escherichia coli BL21(DE3) cells transformed with KES23458 inside a pETcc2 vector in LB medium containing ampicillin (100 μg/ml) at 37°C. When the optical density at 600 nm (OD_600_) reached 0.6 to 1.0, the culture was induced by the addition of IPTG (isopropyl β-d-1-thiogalactopyranoside; 1 mM final concentration) and further incubated at 37°C for 18 h. The cells were isolated by centrifugation in a Beckman Avanti J-E centrifuge (3,000 × *g*; 20 min), and the supernatant was discarded. The pellet was resuspended in imidazole-potassium phosphate buffer (5 mM imidazole, 10 mM phosphate; pH 7.5), and cell lysis was achieved by homogenization at 20,000 lb/in^2^ using an Avestin Emulsiflex-C3 apparatus. Cellular debris was precipitated by centrifugation (40,000 × *g*; 45 min), and the supernatant was passed over a HiTrap chelating HP column (GE Healthcare, Little Chalfont, United Kingdom) on an Åkta fast protein liquid chromatography (FPLC) system (GE Healthcare). Protein was eluted with an increasing concentration of imidazole (5 to 500 mM), and the fractions containing protein were transferred into potassium phosphate buffer (10 mM; pH 7.5), concentrated by centrifugation (10,000 molecular weight cutoff [MWCO]; GE Healthcare), and further purified by gel filtration (Superdex 200; GE Healthcare) in the same phosphate buffer. Purity was estimated to be >95% by SDS-PAGE.

A gene encoding KES23460, codon optimized for expression in E. coli, was ordered in the expression vector pRSetB from Life Technologies (Germany). E. coli BL21(DE3) was transformed with pRSetB containing the gene encoding KES23460, and overexpression and purification of KES23460 was achieved in the same manner as described for KES23458.

### Protein crystallization.

The concentrated protein (8 mg/ml) was used to set up initial crystallization trials (a total of 384 experiments, with the JCSG+ screen at 8°C and 20°C and the PACT [pH, anion, and cation testing] screen and an in-house pH-versus-salt gradient screen both at 20°C). The crystallization droplets consisted of 200 nl protein (8 mg/ml in 10 mM potassium phosphate buffer [pH 7.5]) and 200 nl crystallization cocktail, equilibrated against 50 μl of the cocktail in SD-2 sitting drop plates (Molecular Dimensions, Suffolk, United Kingdom). The best crystals grew from midweight polyethylene glycols (PEG) in the presence of magnesium (e.g., 10% [wt/vol] PEG 4000, 0.2 M magnesium chloride, and 0.1 M Tris-HCl [pH 7]). Crystals appeared within hours and grew to full size (70 μm by 70 μm by 200 μm) within days. Crystals were selected and cryoprotected by the addition of reservoir solution supplemented with 20% glycerol and flash-cooled in N_2_.

### Data collection and structural determination.

For an initial native data set, 360° of data were collected from a single crystal (1° oscillations) to yield a complete X-ray data set to 1.33-Å resolution; a later data set was collected to 1.24 Å, and this higher-resolution data set is summarized below in Table 3. XDS (25) was used to index the reflections, and SCALA (26) was used to scale the data. The structure was solved by molecular replacement using Phaser (27) and was based on the ω-amino acid:pyruvate aminotransferase from Pseudomonas putida (PDB accession number 3A8U) (28). A single protomer was placed in the asymmetric unit, rebuilt manually using Coot (29), and refined using Refmac (30). Details of the data collection and refinement are shown in Table 3.

All data sets were collected at the MX-2 beamline of the Australian Synchrotron. Additional data sets beyond the initial native data set were processed in the manner described above (but were solved using the native structure, rebuilt with Coot, and refined with Refmac).

### Phosphoprotein staining.

Sample preparation for ProQ Diamond phosphoprotein staining was carried out following the manufacturer's protocol (Thermo Fisher Scientific). Purified KES23458 (40 μl, 5 mg/ml) with or without gabaculine (20 μl, 10 mg/ml), as well as crude cell lysate and bovine serum albumin (20 μl, 10 mg/ml) were analyzed. Staining was carried out following the manufacturer's guidelines, and gel visualization was carried out using a Typhoon Trio system (GE Healthcare), with an excitation wavelength of 532 nm, an emission wavelength of 555 nm, and a 500-V photomultiplier tube.

### Assaying for activity.

Activity for KES23458 was assessed using enzyme-coupled dehydrogenase assay mixtures typically comprising substrate (5 mM final concentration in potassium phosphate buffer [100 mM; pH 10]), cosubstrate (pyruvate [0.5 mM final concentration] or α-ketoglutarate [α-KG; 0.25 mM final concentration]), NAD (1.25 mM final concentration; ε_340_ of 6,220 M^−1^ cm^−1^), dehydrogenase (0.035 U of alanine dehydrogenase [ADH], where 1 U corresponds to the amount of enzyme which converts 1 μmol l-alanine per minute at pH 10.0 and 30°C, or 0.925 U of glutamate dehydrogenase [GDH], where 1 U will reduce 1 μmol of α-ketoglutarate to l-glutamate per min at pH 7.3 at 25°C) in potassium phosphate buffer (100 mM; pH 7 to 10). Following the addition of the transaminase enzyme, UV absorbance at 340 nm was recorded at 28°C for 30 min on a SpectraMax M2 UV photospectrometer (Molecular Devices, Australia). For amination reactions, a typical assay mixture comprised substrate (5 mM), alanine (0.25 mM), NADH (0.125 mM), (NH_4_)_2_SO_4_ (10 mM), ADH (0.035 U), and transaminase. The reaction was followed by recording the hypochromic shift at 340 nm in a UV spectrophotometer.

Thermotolerance was determined by residual activity measurements. A potassium phosphate solution of the transaminase was incubated over a range of temperatures (23 to 68°C) for 5 min, centrifuged briefly to remove precipitate, and transferred to a UV spectrophotometer for testing using the transaminase assay described above.

**FIG 1:**
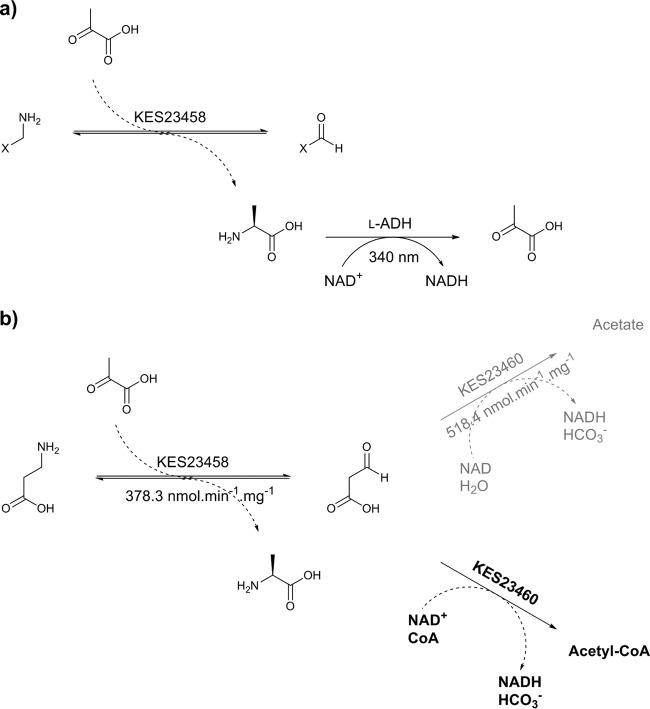
(a) Alanine dehydrogenase-coupled UV assay for transaminase activity. The transaminase catalyzes the amine transfer from the substrate to pyruvate (cosubstrate), producing alanine as a coproduct. The dehydrogenase then catalyzes the oxidative deamination of alanine utilizing a cofactor, NAD^+^ (NAD), which is concurrently reduced to NADH. Formation of NADH can be detected by UV photospectrometry as a hyperchromic shift at 340 nm. (b) Analogous assays performed with KES23460. In this case the transaminase reaction shown is the β-alanine/pyruvate transamination reaction. If CoA is present, malonate semialdehyde produced by transamination is then converted by KES23460 to acetyl-CoA in an NAD^+^ dependent reaction (shown in bold). Without CoA present malonate semialdehyde is converted to acetate and bicarbonate (shown in gray). Both of these reactions require the conversion of NAD^+^ to NADH, which can be followed by UV photospectrometry at 340 nm. Based on spectrophotometric data, and the value of acetyl-CoA as a metabolic intermediate, the CoA-dependent pathway is likely to be the physiologically relevant reaction for these genes.

Determination of KES23460 activity was achieved using an analogous transaminase-coupled assay (Fig. 1b). KES23458 was added to substrate (5 mM), pyruvate (0.5 mM), cofactor (NAD or NADH; 1.25 mM), and KES23460 in potassium phosphate buffer (100 mM; pH 9). Changes in absorbance at 340 nm were monitored for up to 30 min.

### Mass spectrometry.

Protein size was confirmed by mass spectrometry using an Agilent 1100 series LC with a TOF (time of flight) mass spectrometer and Agilent Masshunter workstation (v. A.02.02). A 5-μl volume of purified KES23458 (1.85 mg/ml) was injected into a mobile phase of water (plus 0.1% formic acid) and acetonitrile (plus 0.1% formic acid) (50:50 [vol/vol]; flow rate, 0.8 ml/min) and analyzed in positive electrospray ionization mode for 2 min. The mass spectrometer was set to detect ions with an *m*/*z* between 200 and 3,000 with a fragmentor voltage of 225 V. The mass spectrum was deconvoluted using Agilent Protein TOF confirmation software (v. 1.01.00), with a limited *m/z* range of 600 to 3,000 and a signal/noise threshold of 30, to yield H^+^ adducts of monomeric KES23458 with or without PLP. A molar excess of sodium borohydride was used to ensure complete reduction of PLP.

### Docking studies.

The volume of the active site pocket was calculated using the CASTp server (31).

Models of KES23458 were prepared in Accelrys Discovery Studio v. 3.5, using the PDB file from the experimentally derived structure as a starting point and removing small molecules and water by using the clean protein tool. Models for the β-alanine:PLP and 12-aminododecanoic acid:PLP external aldimine intermediates were created in Accelrys Discovery Studio v. 3.5 and prepared for docking using the full minimization tool in Discovery Studio v. 3.5 with the default settings (CHARMm force field). Atomic charges were calculated using Accelrys Materials Studio v. 8.0 with the QEq method, and the substrates were manually docked into the active site. The protein models were solvated in a TIP3P octahedral solvent box with a 12-Å periodic boundary and charge neutralized by the addition of Na^+^ ions.

Initial minimization on both systems was performed in Amber (v. 14) over 10,000 steps under a constant pressure of 10^5^ Pa. Bond lengths for bonds involving hydrogen were constrained in Shake, and force evaluation of these bonds was not performed. Molecular dynamics (MD) simulations (200 ns) with a step size of 0.002 ps were performed at 310 K and 10^5^ Pa pressure with a 2-ps relaxation time. Simulations were visualized in VMD (v. 1.9.2), and root mean square deviation (RMSD) analysis was performed using the RMSD Trajectory tool. Analysis was conducted on 150 ns of the simulation, removing the first 50 ns to ensure the systems were equilibrated.

### Saturation mutagenesis protocol.

Mutagenesis of the DNA from Pseudomonas sp. AAC encoding KES23458 was achieved using a QuikChange Lightning Multi site-directed mutagenesis kit (Agilent). An annealing temperature of 60°C was selected, and 1.5% dimethyl sulfoxide was added to the reaction mixture (R414NDT mutagenic primer, 5′-GAAGGAAGGCTTCTACGTGNDTTTCGGCGGCGACACCCTGCAG-3′). All other elements of the protocol were as described in the manufacturer's guidelines. The CGC codon, which encodes arginine, was mutated using a degenerate NDT codon (32). The resulting DNA was transformed into E. coli BL21 λDE3 cells by electroporation and plated on LB agar containing ampicillin (LB-Amp; 100 μg/ml ampicillin).

Thirty-five individual colonies (>95% coverage) were picked from the LB agar plates. An additional E. coli colony containing the wild-type (WT) DNA was also picked and added as a positive control, for a total of 36 colonies. Each colony was used to inoculate 200 μl LB containing ampicillin (100 μg/ml) in a 96-well plate. This plate (the “mother plate”) was incubated overnight at 37°C with shaking (700 rpm). Ten-microliter aliquots were taken from each well and added to 1 ml LB-Amp in a 96-deep-well block (the “daughter block”). The daughter block was incubated at 37°C for 4 h with shaking (1,000 rpm), while the mother plate was transferred to storage at 4°C. IPTG was added to each well (1 mM, final concentration) of the daughter block, which was then further incubated overnight at 37°C with shaking.

The daughter block was centrifuged at 3,000 × *g* for 20 min, and the supernatants were discarded. To each well, 10 μl BugBuster (10× reagent; EMD Millipore) and 90 μl potassium phosphate buffer (100 mM; pH 7.5) were added, and the block was incubated for 1 h at 4°C and finally centrifuged at 4,750 × *g* for 45 min. The 100 μl of supernatant from each well was transferred to the corresponding well in a new 96-well “stock” plate and stored at 4°C.

For activity screening, 5 μl supernatant from each well of the stock plate was added to the standard assay mixture described previously, where the substrate was a saturated solution of 12-aminododecanoic acid. UV absorbance at 340 nm was monitored for 1 h at 1-min intervals. Hits were identified as those exhibiting a hyperchromic absorbance change. For each hit, a new 10-ml culture of LB containing ampicillin (100 μg/ml) was inoculated using the corresponding culture from the mother plate and incubated at 37°C overnight with shaking. DNA was isolated from the cells by using a Bioline Isolate II plasmid minikit, and DNA was sequenced by Macrogen Inc. (Seoul, South Korea).

### Protein structure accession numbers.

The models and data from our study have been deposited in the Protein Data Bank under accession codes 4UHO (1.24 Å), 4UHN (phospho-His), and 4UHM (1.33 Å).

## RESULTS AND DISCUSSION

KES23458 was previously shown to catalyze the transamination of two substrates, β-alanine and 12-aminododecanoic acid. Given the large difference in sizes between its hypothetical natural substrate and the observed promiscuous substrate (β-alanine and 12-aminododecanoic acid, respectively), we anticipated that the enzyme could potentially catalyze all the homologous substrates in between (i.e., C_4_ to C_11_) to produce a range of polyamide building blocks. As such, we sought to further characterize this enzyme, both in terms of promiscuity and its physiological role with the Pseudomonas sp. strain AAC.

### Substrate specificity and characterization of KES23458.

The promiscuity of the transaminase was assessed using a well-established, dehydrogenase-coupled assay (Fig. 1a) with a broad range of substrates (50 compounds [see Fig. S1 in the supplemental material]). KES23458 was found to be highly promiscuous, exhibiting activity with 39 of the compounds tested. These included a broad range of different amines, including α-, β-, and ω-amino acids, aminated cyclic substrates, and a selection of diamines, as well as aldehydes and ketones (substrates along with their specific and relative activities are shown in Table 1). Unfortunately, as alanine dehydrogenase is strongly inhibited by low millimolar concentrations of pyruvate (in the same manner as other similar dehydrogenases) (33), it was not possible to determine steady-state kinetics by this method. However, it was possible to obtain specific activity data for the transaminase and begin to understand its substrate preferences. As anticipated, the enzyme was observed to catalyze the transamination of ω-aminoalkanoates ranging in length from C_3_ to C_12_, with specific activities ranging from 126.1 (C_12_) to 699.7 (C_8_) nmol · min^−1^ · mg^−1^, although no obvious correlation between substrate size and specific activity was observed (see Fig. S2 in the supplemental material). In addition, the enzyme was also able to turn over a range of aliphatic α/ω-diamines with specific activities ranging from 24.7 (C_3_) to 679.9 (C_10_) nmol · min^−1^ · mg^−1^. Like the ω-amino acids, these diamines have applications in the production of polymers, notably 6,6-hexamethylenediamine for the production of nylon-6,6, and as such KES23458 is a promising biocatalyst for the production of a range of nylon building blocks. As with the amino acid substrates, no clear correlation between the size of the diamine substrate and enzyme activity was observed (see Fig. S2).

**TABLE 1:** Specific and relative activities for a range of amine donors and acceptors

Substrate category and name^*a*^	Sp act (nmol/min/mg)	Relative activity^*b*^ (%)
Amine donors		
ω-Aminoalkanoates		
Glycine	9.9 ± 2.4	1.4
β-Alanine	378.3 ± 7.4	54.1
4-Aminobutyric acid	635.4 ± 9.9	90.8
5-Aminopentanoic acid	368.4 ± 1.9	52.7
6-Aminohexanoic acid	437.6 ± 1.3	62.5
7-Aminoheptanoic acid	482.1 ± 2.5	68.9
8-Aminooctanoic acid	699.7 ± 9.9	100.0
12-Aminododecanoic acid	126.1 ± 2.5	18.0
Diamines		
1,3-Diaminopropane	24.7 ± 0.7	3.5
Putrescine	267.0 ± 1.7	38.2
Cadaverine	603.0 ± 0.8	86.2
1,6-Hexamethylenediamine	388.2 ± 1.7	55.5
1,7-Heptanediamine	346.2 ± 2.5	49.5
1,8-Octaneamine	279.4 ± 2.5	39.9
1,9-Nonanediamine	212.6 ± 2.5	30.4
1,10-Decanediamine	679.9 ± 7.4	97.2
Amino acids		
l-Ornithine	4.9 ± 0.3	0.7
d-Ornithine	27.2 ± 0.3	3.9
l-Lysine	2.5 ± 0.2	0.4
d-Lysine	61.8 ± 0.4	8.8
l-Arginine	4.9 ± 0.4	0.7
d-Arginine	ND^*c*^	ND
l-Glutamic acid	ND	ND
Citrulline	ND	ND
Other amine donors		
*N*-Acetyl-l-Ornithine	ND	ND
4-Amino-2-(*S*)-hydroxybutyric acid	336.3 ± 2.5	48.1
5-Aminolevulinic acid	ND	ND
Serinol	9.9 ± 0.2	1.4
d/l-Aminoisobutyric acid	435.2 ± 2.5	62.2
Taurine	7.4 ± 0.5	1.1
Creatine	ND	ND
3-Aminocyclohexanoic acid	12.4 ± 0.7	1.8
3-Aminoheptanoic acid	2.5 ± 0.5	0.4
(*S*)-2,4-Diaminobutyric acid	ND	ND
β-Homoleucine	ND	ND
2,6-Diaminopimelate	ND	ND
Aminomethylphosphonic acid	ND	ND
2-Aminoindane	24.7 ± 0.6	3.5
2-Methylbenzylamine	54.4 ± 4.9	7.8
Isopropylamine	232.4 ± 1.3	33.2
Cyclohexylamine	42.0 ± 0.4	6.0
6-Aminohexanol	489.6 ± 2.4	70.0
Amine acceptors		
Propionaldehyde	202.0 ± 7.2	68.3
Butyraldehyde	295.8 ± 7.2	100.0
Heptanal	21.6 ± 1.8	7.3
Octanal	24.1 ± 2.1	8.1
Dodecanal	40.9 ± 19.2	13.8
Glyceraldehyde	101.0 ± 2.4	34.1
Cyclohexanone	ND	ND
HMF	40.9 ± 1.1	13.8

a For each amine donor substrate, the acceptor used was pyruvate (assay conditions included potassium phosphate buffer at 100 mM, pH 10). For each amine acceptor substrate, the donor used was L-alanine (assay conditions included potassium phosphate buffer at 100 mM, pH 8).

b Relative activities are in comparison with 8-aminooctanoic acid for the various amine donors and with butyraldehyde for the amine acceptors.

c ND, activity not determined.

The transaminase also exhibited activity with monofunctionalized aliphatic aldehydes, such as butyraldehyde, octanal, and decanal, with the highest specific activity observed for butyraldehyde (295.8 nmol · min^−1^ · mg^−1^). The other aldehydes had significantly lower activities; heptanal, octanal, and decanal showed specific activities of 7, 8, and 14%, respectively, relative to butyraldehyde. However, this may have been the result of lower effective concentrations of these compounds due to their low aqueous solubilities. Again, as with the amino acids and diamines, there was no clear correlation between the length of the aliphatic chain of the substrate and enzyme activity (see Fig. S2 in the supplemental material). Finally, KES23458 showed activity against the biologically and industrially relevant compounds 5-hydroxymethylfurfural (HMF; specific activity, 40.9 nmol · min^−1^ · mg^−1^) and glyceraldehyde (specific activity, 101 nmol · min^−1^ · mg^−1^), both of which have been identified by the U.S. Department of Energy as biorefinery product opportunities from carbohydrates (34).

In addition to a broad substrate range, the thermotolerance of the enzyme was assessed by assaying residual activity after heat treatment. Using this method, 50% residual activity was observed after preincubation at 63°C, and only 5% residual activity was observed after a 68°C incubation (see Fig. S3A in the supplemental material). Another estimate of the thermostability of the enzyme was obtained by differential scanning fluorimentry (DSF), in which the fluorescence of an environmentally sensitive dye is used to follow the unfolding of a protein. By this method, the melting temperature was determined to be 58°C (see Fig. S3B).

### Transaminase crystallization and structural elucidation.

We undertook crystallographic analysis in an effort to understand the structural basis for the broad substrate specificity exhibited by the transaminase. The transaminase was expressed and purified by affinity chromatography as described above and then further purified by gel filtration (see Fig. S4 in the supplemental material). Based on its elution profile, the transaminase was predicted to exist as a dimer in solution (described in more detail in Materials and Methods), consistent with other known structures of ω-transaminases (13, 14). The protein was concentrated to 8 mg/ml, and initial crystallization trials were carried out at 8 and 20°C. Crystals were observed within hours in a number of wells containing polyethylene glycol, and although most of the crystals were thin plates, chunky rectangular prisms (see Fig. S5 in the supplemental material), which diffracted X-rays to better than 1.4 Å, were observed under optimal conditions (described in Materials and Methods). Despite having different morphologies, most crystals were found to be in the I222 space group with approximately the same cell dimensions. The molecules in the initial, well-diffracting crystals appeared to pack in a way which would hinder access to the active site. Cocrystallization trials with 8-aminooctanoic acid, 12-aminododecanoic acid, d/l-3-aminoisobutyric acid, β-alanine, and a common TA suicide inhibitor, gabaculine, in both broad (initial) screens and fine screens under the initial conditions also failed to yield structures with any obvious electron density for a small molecule. In addition, our lack of success with substrate soaking experiments may have been the result of occlusion of the active site by the crystal packing.

**FIG 2:**
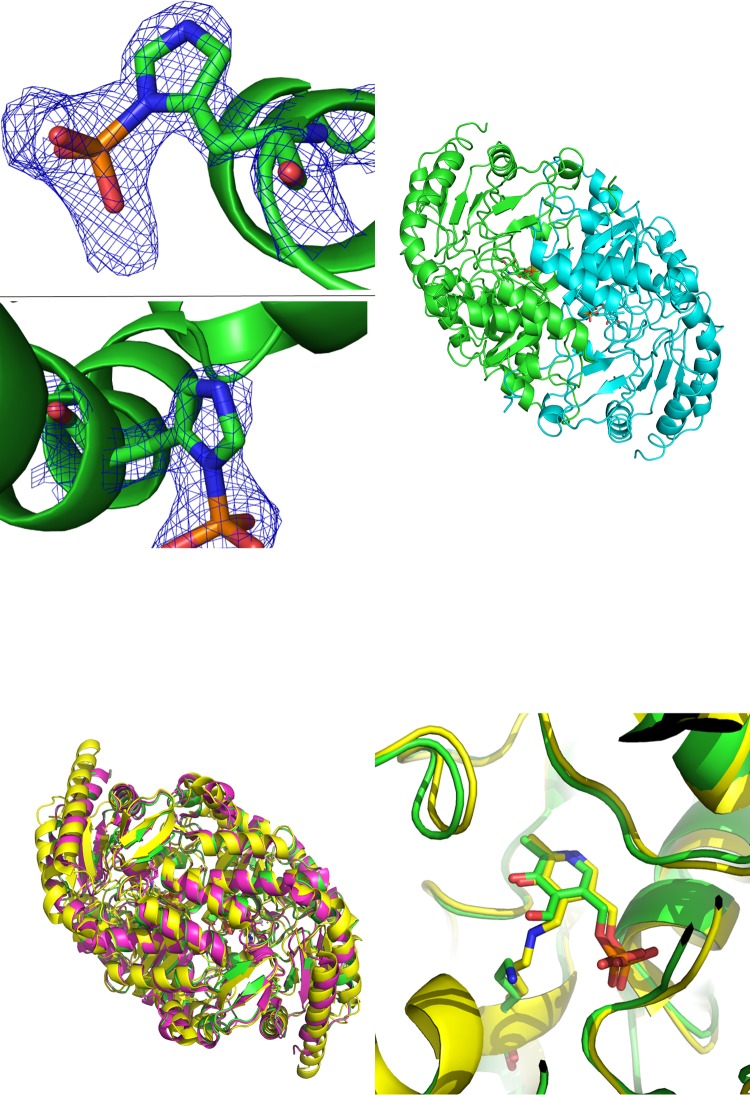
(Upper left) 2mFo-DFc electron density maps for the phosphohistidine groups observed in the X-ray diffraction data for KES23458 at 1.5 σ. Diagrams show His_31_ (top) and His_360_ (bottom). (Upper right) Dimeric form of KES23458. One monomeric subunit is shown in green and the other in cyan, with PLP shown in each active site. (Lower left) Overlay for KES23458 from Pseudomonas sp. strain AAC, shown in green. The protein 4A6T from Chromobacterium violaceum is shown in yellow. The protein 4B98 from Pseudomonas aeruginosa is shown in magenta. (Lower right) The two proteins, KES23458 and 4A6T, overlaid, with the free PLP and Lys_288_ from KES23458; the internal aldimine from 4A6T is represented by sticks.

The structure revealed a dimeric α/β-fold protein with a total surface area of about 37,000 Å^2^ and a buried surface of 2,880 Å^2^, according to information in the PISA database (35), confirming the gel filtration results. On comparison with other available ω-transaminase structures from Pseudomonas aeruginosa and Chromobacterium violaceum (87% and 29% sequence identity to KES23458, respectively; PDB accession numbers 4B98 and 4A6T), KES23458 was observed to be similar, with backbone RMS values of 0.2 Å and 1.3 Å, respectively (Fig. 2, bottom left structure), illustrating that despite low sequence homology, overall structural conservation remains high in this enzyme class.

The dimer contained two long active sites; each was a large, solvent-accessible cavity comprised of residues from both monomers (Fig. 2, upper right structure), with a Connolly surface volume of 1,771 Å^3^. This is sufficient to completely enclose substrates as large as 12-aminododecanoic acid and in part rationalizes the broad substrate specificity observed for the enzyme. It is unclear, however, why such a large active site cavity has evolved when the physiological substrate and cosubstrate are β-alanine and pyruvate, respectively, or how selective ingress of the physiological substrate is achieved. It is possible that this protein may perform more than one physiological function within the organism, which is consistent with the high levels of activity observed with multiple substrates.

Examination of the electron density in the presumed active site showed a close analogue of PLP (denoted PLP′) bound where one would expect to find the native cofactor. This density could not be resolved but was atypical for a molecule of PLP. It is unclear why native PLP was not seen in these very-high-resolution data sets, as additional experiments were set up with fresh PLP added to the protein and yielded the same anomalous result. Each PLP′ was coordinated to Gly_120_, Ser_121_, Tyr_153_ Asp_259_, Lys_288_, and Thr_327_′ (where the prime sign denotes that the residue is from the other monomer of the transaminase) via hydrogen bonding, and although PLP is typically bound to the protein by a covalent bond (an internal or external aldimine) (36) with an active site lysine residue, aldimine-bound PLP was not observed in the structure. However, when overlaid against other transaminase crystal structures (PDB accession numbers 4B98 and 4A6T) (13), a homologous lysine residue (Lys_288_) lying 2.4 Å from the cofactor was apparent, and is likely to fulfil that role in KES23458 (Fig. 2, bottom right structure). Further analysis of the enzyme-cofactor complex was carried out by mass spectrometry, which confirmed the identity of the full-length peptide minus the N-terminal methionine (see Fig. S5 and the sequence detailed in the supplemental material). Both the apo- and holo-forms of the protein were observed in the spectrum, with the mass gain corresponding to aldimine-bound unmodified PLP. No additional peaks which could correspond to PLP analogues were observed in the spectrum. Treatment with sodium borohydride was also used to reduce the aldimine and bind the cofactor irreversibly. The resultant mass spectrum showed a deconvoluted peak *m/z* of 50,399, corresponding to the reduced form of KES23458 and PLP, supporting the presence of PLP in the protein (rather than an analogue), as well as confirming aldimine bond formation. As such, we suggest that the PLP′ analogue observed in the crystal structures is an artifact of the crystallization process, rather than a novel PLP-like cofactor.

The protein was found to contain phosphorylated histidine residues at positions 31 and 360 (Fig. 2, upper left panel structures). Studies of histidine phosphorylation are less extensive than for other amino acids, such as serine, threonine, and tyrosine, as a consequence of the acid lability of the phosphate group (37). However, protein phosphorylation by histidine kinases is known to be important in prokaryotic signal transduction (38), and an increasing number of histidine kinase activities in higher eukaryotes have been identified (39, 40). The acid lability of phosphohistidine also hindered mass spectrometry characterization, so phosphoprotein staining was used to confirm phosphorylation of KES23458. The transaminase was treated with gabaculine to irreversibly bind the PLP and prevent reformation of an internal aldimine complex with Lys_288_. The protein was then boiled to remove the gabaculine:PLP complex, and visualization of the SDS-PAGE gel using fluorescence (detailed in Materials and Methods) confirmed that despite removal of the phosphorylated cofactor, the protein still contained phosphate, supporting the X-ray data. Although the role of this posttranslational modification remains unclear, and it may simply be an artifact of heterologous expression, phosphorylation of histidine residues at the surface of KES23458 is apparent, and under certain crystallographic conditions these unusual moieties are retained and observed.

### Physiological role for KES23458.

Interestingly, the specific activity for the proposed natural substrate, β-alanine, was only 54% of the activity measured for 8-aminooctanoic acid, which was the preferred substrate under the assay conditions. This observation, together with the large active site, prompted further investigations to probe the physiological role of the transaminase. Attempts to knock out the KES23458 gene from Pseudomonas sp. strain AAC were unsuccessful (using the Gene Bridges K006 kit, designed for E. coli). However, analysis of the genetic organization surrounding the gene suggested that it may form a bicistronic operon with a putative dehydrogenase protein, KES23460. KES23460 was successfully cloned, expressed, and characterized. Based on the peptide sequence of the dehydrogenase, the enzyme was putatively assigned the function of methylmalonate semialdehyde dehydrogenase, which could be accessed by the transamination reaction of aminoisobutyrate. However, because KES23458 had exhibited a wide substrate scope, the activity of the dehydrogenase was tested with a number of substrates (shown in Table S1 in the supplemental material). Substrates were selected based on prior activity with KES23458 and were assayed with the dehydrogenase by coupling it to KES23458 as well as the oxidized and reduced forms of both NAD and NADP cofactors. Absorbance at 340 nm was observed, anticipating that the aldehydes produced by transamination would be further converted by the dehydrogenase and that the concurrent oxidation or reduction of the nicotinamide cofactor could be monitored at 340 nm. Unlike the transaminase, the dehydrogenase was shown to be more selective, having significant activity with only one of the substrates tested, malonic acid semialdehyde, which formed following the transamination of β-alanine.

Based on previous investigations with malonate semialdehyde dehydrogenases (41–44), KES23460 was further investigated to confirm its function. In keeping with previous findings for IolA (44% sequence identity to KES23460), a methylmalonate semialdehyde dehydrogenase from Bacillus subtilis, KES23460, was shown to facilitate the oxidative conversion of malonic acid semialdehyde both with and without CoA. In the presence of CoA, the transthioesterification step was observed to be significantly faster (approximately 10-fold) than the hydrolysis when no CoA was present, and this was also consistent with previous findings (44). It is plausible, based on the specificity of the dehydrogenase, that KES23458 and KES23460 form a β-alanine catabolic pathway in which malonic acid semialdehyde is produced from β-alanine by KES23458-mediated amine transfer and then converted to the common metabolic intermediate acetyl-CoA in an NAD-dependent oxidation by KES23460. The reduction of acetyl-CoA by KES23460 using NADH was also tested, but it could not be detected under the conditions tested; based on the rates we observed, we concluded that this operon appears to form a β-alanine catabolic pathway within Pseudomonas sp. strain AAC, producing acetyl-CoA.

### Molecular dynamics study of KES23458 with β-alanine and 12-aminododecanoic acid.

To improve our understanding of the structure-activity relationship of KES23458, 200-ns MD simulations were carried out whereby β-alanine and 12-aminododecanoic acid were separately docked in the active site of the enzyme in an external aldimine conformation. The substrates were manually orientated in the active site, using the electron density from PLP′ as a guide and consistent with other transaminase structures, with the aromatic nitrogen of the PLP moiety in close proximity to Asp_259_. The simulations were carried out under identical conditions in order to compare changes in protein conformation and substrate binding (as described in Materials and Methods). Backbone RMSD analysis was performed to determine the equilibration time of the system and this was discarded from analysis (see Fig. S7 and further descriptions of materials and methods in the supplemental material).

**FIG 3:**
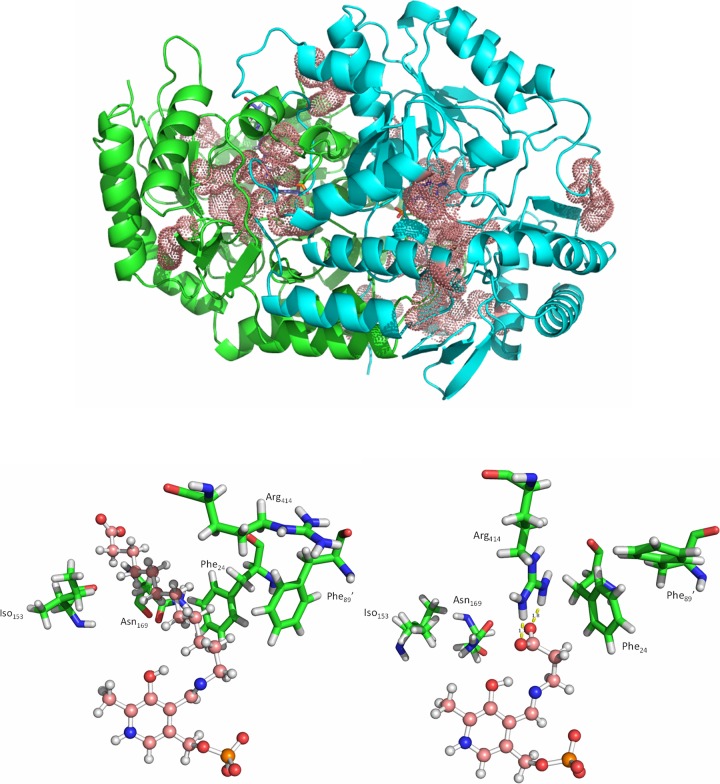
(Top) The dimeric form of KES23458, with monomers shown in green and cyan. Residues which were identified as having a difference in average backbone RMSD of >1.5 Å between the two MD simulations are represented by pink dots. Note that in each case the substrate was docked into the same (green) monomeric subunit, and PLP alone was docked into the cyan monomeric subunit. (Bottom) Visualization of the active site when 12-aminododecanoic acid (structure on left) and β-alanine (structure on right) were docked independently. In each case, the substrate:PLP complex is shown in pink in a ball-and-stick representation. Residues which were observed to change conformation most significantly are shown in green.

Analysis of the MD simulation results exemplified the plasticity of the active site of KES23458. The average backbone RMSD for each residue was calculated over 150 ns in each system. The average value for each residue was then compared between the two systems, and the largest observed changes (>1.5 Å) were mapped onto the original crystal structure (Fig. 3, top diagram). Thirty-one residues were identified whose average backbone RMSD was >1.5 Å different between the two simulations, i.e., these 31 residues showed the biggest differences in position when binding one substrate or the other. While the residues were largely located around the PLP binding sites, loops around the entrance to the active site, as well as distant loops on the surface of the protein were also observed to adopt different conformations depending on the substrate bound in the system. It was also observed that many of the conformational changes observed (19 of 31) were in the active site of the monomer which contained PLP but no substrate.

On closer examination of the 12-aminododecanoic acid simulation, significant structural rearrangements were apparent, with notable movements from hydrophobic residues, including Phe_24_, Phe_89_′, Iso_166_, and Asn_169_ (1.4- to 3.5-Å differences in RMSD between the two simulations) which combined with others to form a large surface around the substrate. These movements also acted to shield the substrate from more polar residues, such as Tyr_153_ and Arg_414_ (0.8- and 2.6-Å difference in RMSD, respectively), which rotated away from the active site (Fig. 3, bottom left and right diagrams). Toward the carboxylate end of the substrate, close to the surface of the protein, the active site expands and the substrate appears to move randomly in the solvent-accessible space. Based on the crystal structure, it was initially anticipated that Lys_171_ may form a hydrogen bond with the α-carboxylic acid terminus of the substrate, and although this loop did undergo significant conformational changes, analysis revealed that this bond was only intermittently formed and no stable interactions were observed between the substrate carboxylic acid and the protein. In contrast, in the β-alanine system, Arg_414_ appeared to be catalytically relevant, as it was ideally positioned to stabilize the carboxylate terminus of β-alanine through a stable electrostatic interaction (Fig. 3, bottom right). This interaction positions the substrate ideally for aldimine formation with PLP and would improve subsequent stabilization of the aldimine intermediate in the active site. This supports the observation that β-alanine activity is significantly higher than that of 12-aminododecanoic acid, as well as the notion that the active site evolved toward β-alanine catalysis. Alignment of the structure of KES23458 with other known ω-transaminases (PDB accession numbers 3A8U, 3FCR, and 3I5T) revealed that Arg_414_ aligns with Arg_417_ from other structures, the so-called “flipping arginine,” responsible for dual-substrate specificity (45). To further elucidate the role of Arg_414_, the residue was subjected to saturation mutagenesis. An NDT codon library which encoded 12 possible amino acids was generated, and 35 transformants were screened (>95% coverage) for activity with 12-aminododecanoic acid. All transformants exhibiting transaminase activity were recovered and DNA was sequenced, and in each case it was found that the protein contained an arginine residue at position 414 exclusively. A number of transformants containing inactive variants were also sequenced and were found to have an Arg_414_ replaced with Ile (ATT), Val (GTT), Gly (GGT), or Asp (GAT) residues. In addition, Arg_414_Lys (AAA codon) was also created, as this was not encoded by the NDT codon but was essential to probe the function of Arg_414_. The five variants were individually overexpressed and purified in the same manner as the WT protein and tested against a small number of additional substrates (β-alanine, 1,3-diaminopropane, putrescine, and cadaverine). Diamines were tested in order to determine whether a reversal in the charge at residue 414 (Arg_414_Asp) could be paired with a reversed charge in the substrate (carboxylic acid to amine), and longer-chain amines were tested to account for the size difference between arginine and aspartic acid. All five variants were successfully purified, but activity was only detectable for the Arg_414_Lys variant, which retained activity against all of the substrates tested except 12-aminododecanoic acid. In each case Arg_414_Lys activity was significantly lower than in the WT enzyme (<20%) (Table 2).

**TABLE 2:** Specific and relative activities for the KES23458 R414K variant compared to the WT^*a*^

Substrate	R414K sp act (nmol/min/mg)	Relative activity (% of WT)
β-Alanine	75.1 ± 1.0	19.9
12-Aminododecanoic acid	ND	ND
Putrescine	38.6 ± 2.0	14.4
Cadaverine	57.9 ± 1.0	9.6
1,3-Diaminopropane	5.1 ± 1.0	20.5

a Note that for each substrate, the acceptor used was pyruvate. Assay conditions are described in Materials and Methods (and included potassium phosphate buffer at 100 mM, pH 10). ND, activity not determined.

These findings are in agreement with previous reports in the literature and indicate that Arg_414_ acts as a “flipping arginine” in KES23458. A positively charged group at position 414 is essential for catalysis with Arg_414_, which is strongly favored at this position. Although replacement with a lysine yielded a functional mutant, activity was greatly reduced for β-alanine (19.9% relative to WT KES23458), and activities with other substrates were impacted more significantly (Table 2).

### Conclusions.

KES23458 was previously identified as a prospective industrial biocatalyst because of its ability to metabolize 12-aminododecanoic acid. The subsequent analyses presented here demonstrate that KES23458 is a highly promiscuous transaminase, catalyzing amine transfers with 39 of the 50 substrates tested. Although this broad substrate range can be partially rationalized due to the sheer size of the substrate binding pocket, molecular dynamics simulations indicated that the enzyme is able to undergo significant structural rearrangements to accommodate different substrates. As is the case for the ω-transaminase family generally, the structure-function relationship of KES23458 is poorly understood, and consequently, there are few examples of rationally designed ω-transaminase variants with significantly improved activity toward nonphysiological substrates (8, 16, 46).

To provide further characterization of the transaminase, additional investigations into the concomitant dehydrogenase (KES23460) from the bicistronic operon containing KES23458 were carried out, and a physiological role for the transaminase was confirmed. In contrast to the transaminase, the dehydrogenase enzyme was highly selective toward malonate semialdehyde, suggesting that the transaminase in fact has a specialized function within a β-alanine catabolic pathway in Pseudomonas sp. strain AAC. The dehydrogenase was also observed to facilitate both CoA-dependent and -independent reactions, in agreement with previous literature findings, although superior rates were observed for the CoA-dependent pathway to produce acetyl-CoA.

Notwithstanding the current limitations for rational engineering of KES23458, the thorough characterization of the enzyme, including an understanding of its apparent specialization toward β-alanine, together with its broader characterization and a high-resolution crystal structure (Table 3), make it an excellent candidate for biocatalyst development. KES23458 shows broad activity for a range of industrially relevant amines and ω-amino acids, all of which are important precursors in polyamide production.

**TABLE 3:** X-ray data

Parameter	Value for protein with PDB accession no.^*a*^:	
	4UHN (PO_4_-His)	4UHO (native)
Space group	I222	I222
Cell dimensions		
*a*, *b*, *c* (Å)	62.9, 119.7, 134.3	63.0, 120.0, 134.2
α, β, γ (°)	90.0, 90.0, 90.0	90.0, 90.0, 90.0
Resolution range (Å)	44.7–2.21 (2.28–2.21)	44.9–1.24 (1.26–1.24)
*R*_merge_	0.267 (1.122)	0.077 (0.628)
*R*_pim_	0.072 (0.299)	0.033 (0.276)
*CC1*/*2*	0.996 (0.843)	0.999 (0.845)
*I*/*σI*	13.3 (3.3)	12.6 (2.8)
Completeness (%)	100 (100)	99.9 (98.2)
Redundancy	14.7 (14.8)	7.2 (7.0)
Refinement		
Resolution (Å)	44.7–2.21	44.9–1.24
No. of unique reflections	24,514	137,317
*R*_work_/*R*_free_ (%)	14.5/18.8	10.5/12.3
No. of atoms	3,595	4,382
Protein	3,377	3,679
PLP/other	16/12	16/67
Water	190	620
*B*-factors (Å^2^)	23.1	12.1
Protein	22.9	9.9
PLP/other	31.2/39.5	10.7/18.0
Water	24.6	24.2
RMSD		
Bond lengths (Å)	0.020	0.010
Bond angles (°)	1.979	1.666

a The numbers in parentheses represent the values in the high-resolution bin for each of the categories.

## Supplementary Material

Supplemental material
