# Supplementary material for: A β-Alanine Catabolism Pathway Containing a Highly Promiscuous ω-Transaminase in the 12-Aminododecanate-Degrading Pseudomonas sp. Strain AAC
Source: Appl Environ Microbiol. 2016 Jun 13;82(13):3846–56. doi: 10.1128/AEM.00665-16 (PMC4907197; doi:10.1128/AEM.00665-16)
Supplement: Supplemental material [file supp_82_13_3846__index.html]

Supplemental material 

# A β-Alanine Catabolism Pathway Containing a Highly Promiscuous ω-Transaminase in the 12-Aminododecanate-Degrading Pseudomonas sp. Strain AAC

## Supplemental material

- Supplemental file 1 -

  Structures for each substrate tested, with specific activity shown below (Fig. S1); chain length versus specific activity (Fig. S2); KES23458 gel filtration trace (Fig. S3); KES23458 thermotolerance by residual activity, triplicate replicates of a differential scanning fluorimetry assay of KES23458 in 10 mM phosphate buffer or in a range of buffers, salts, and pHs (Fig. S4); TA crystal (Fig. S5); mass spectrometry data (Fig. S6); RMSD analysis of KES23458 with β-alanine and 12-aminododecanoic acid docked (Fig. S7); specific activities and relative substrate activities for KES23460 (Table S1).

  PDF, 1.3M
