# Supplementary material for: A β-Alanine Catabolism Pathway Containing a Highly Promiscuous ω-Transaminase in the 12-Aminododecanate-Degrading Pseudomonas sp. Strain AAC
Source: Appl Environ Microbiol. 2016 Jun 13;82(13):3846–56. doi: 10.1128/AEM.00665-16 (PMC4907197; doi:10.1128/AEM.00665-16)
Supplement: Supplemental material [file AEM.00665-16_zam999117220so1.pdf]

Supplementary Materials

Figure S1 – Structures for each substrate tested, with specific activity shown below (nmol.min^{-1}.mg^{-1}).

-aminoalkanoic acids

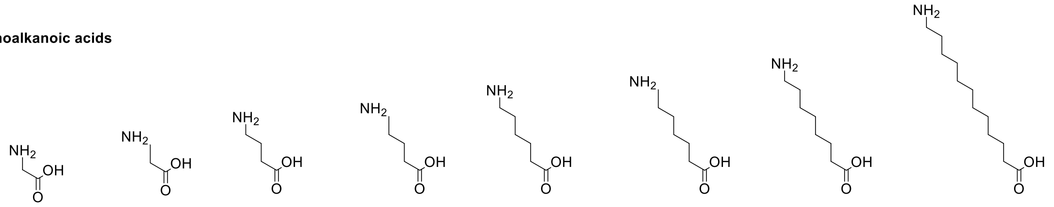

Glycine	□-alanine	C_4	C_5	C_6	C_7	C_8	C_{12}
9.9	378.3	635.4	368.4	437.6	482.1	677.5	126.1

Diamines

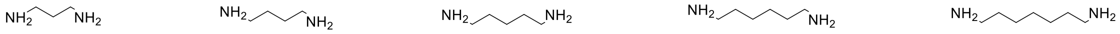

1,3-Diaminopropane 24.7 · Putrescine 272.0 · Cadaverine 603.3 · 1,6-Hexamethylenediamine 383.2 · 1,7-Heptanediamine 341.2

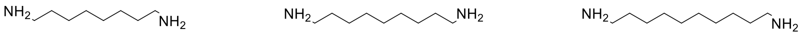

1,8-Octanediamine 284.5 · 1,9-Nonanediamine 210.2 · 1,10-Decanediamine 655.2

Amino acids

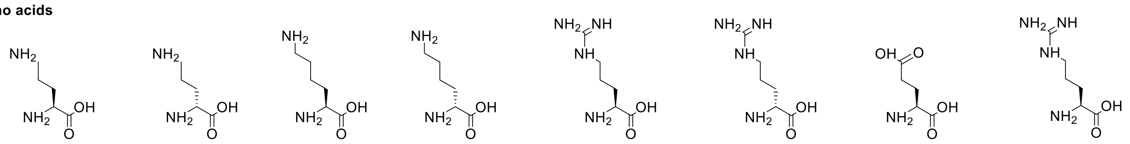

(*L*)-Ornithine	(D)-Ornithine	*L*-Lysine	*D*-Lysine	*L*-Arginine	*D*-Arginine	*L*-Glutamic acid	*L*-Citrulline
4.9	17.3	2.5	61.8	4.9	ND	ND	ND

Others

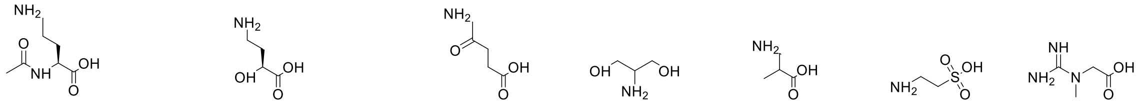

N-Acetyl-*L*-Ornithine	4-amino-2-(*S*)-hydroxybutanoic acid	5-aminolevulinic acid	Serinol	Aminoisobutyric acid	Taurine	Creatine
ND	346.2	ND	2.5	492.0	7.4	ND

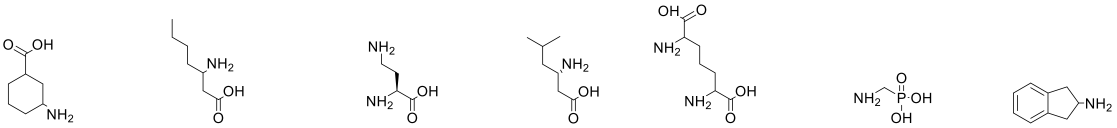

3-aminocyclohexanoic acid	3-aminoheptanoic acid	(*S*)-2,4-diaminobutanoic acid	□-Homoleucine	2,6-diaminopimelate	(aminomethyl)phosphonic acid	2-Aminoindane
12.5	2.5	ND	ND	ND	ND	27.2

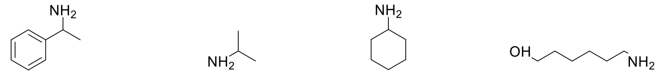

□-Methylbenzylamine	Isopropylamine	cyclohexylamine	6-aminohexan-1-ol
54.4	237.4	42.0	474.7

Aldehydes/Ketones

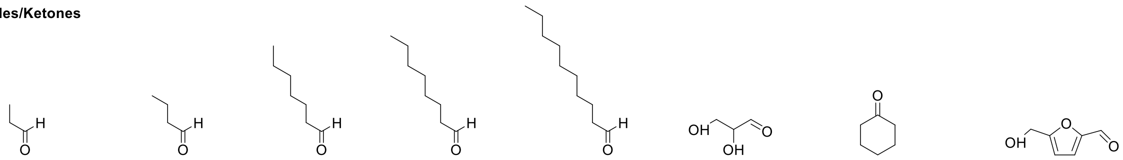

Propionaldehyde	Butyraldehyde	Heptanal	Octanal	Decanal	Glyceraldehyde	Cyclohexanone	5-(Hydroxymethyl)furfural
202.0	295.8	21.6	24.1	40.9	101.0	ND	40.9

Figure S2 – Chain length vs. Specific Activity. SD shown as error bars.

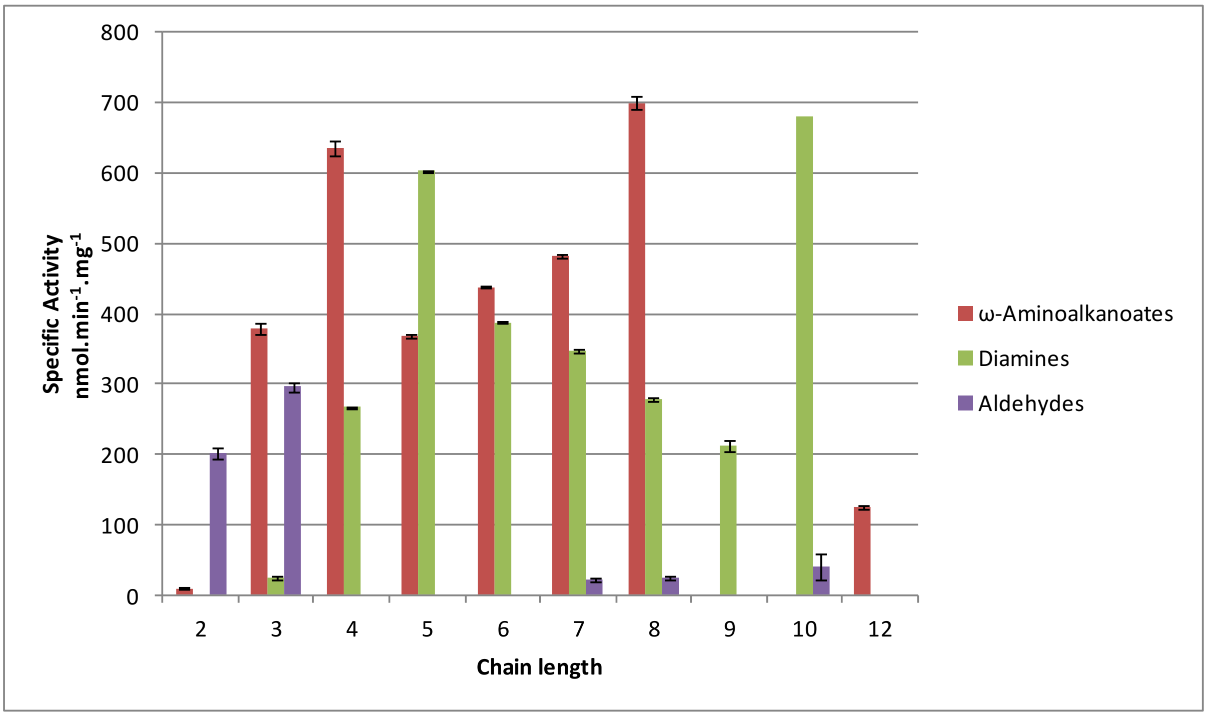

Figure S3 – KES23458 Gel Filtration Trace

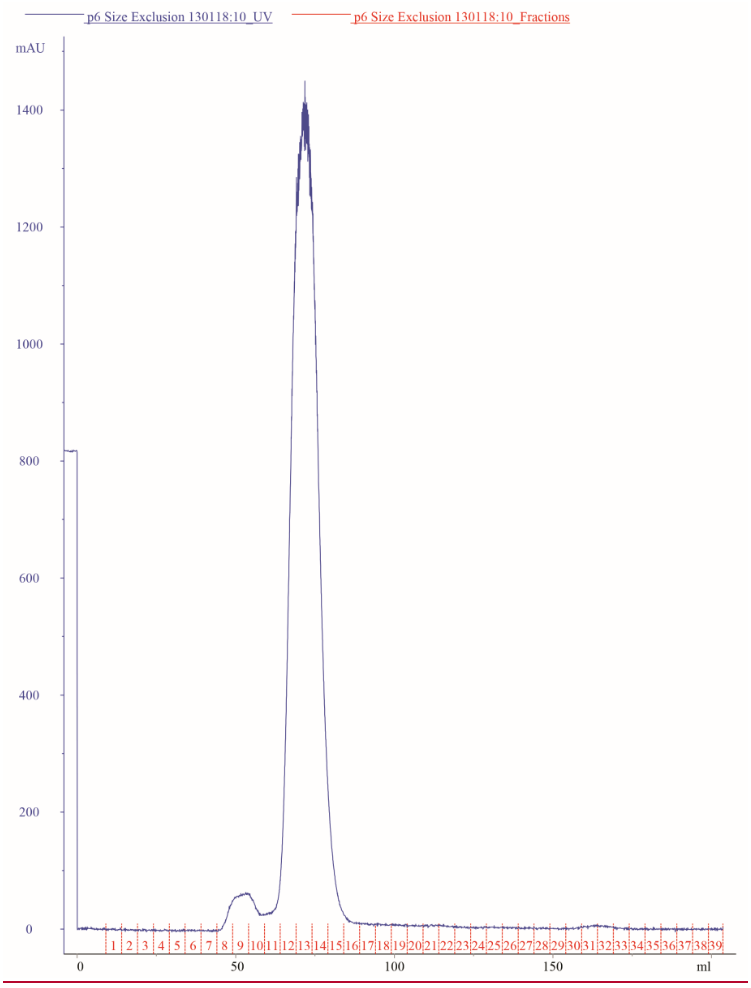

Figure S4 (a) – KES23458 thermotolerance by residual activity. Activity was measured using a standard assay as described in the Experimental section and the data was fitted to a sigmoidal curve with equation $f(T) \; = 100/(1 + e^{(T-T50)})$ where T is the temperature and $f(T)$ is the residual activity (%) at a given T.

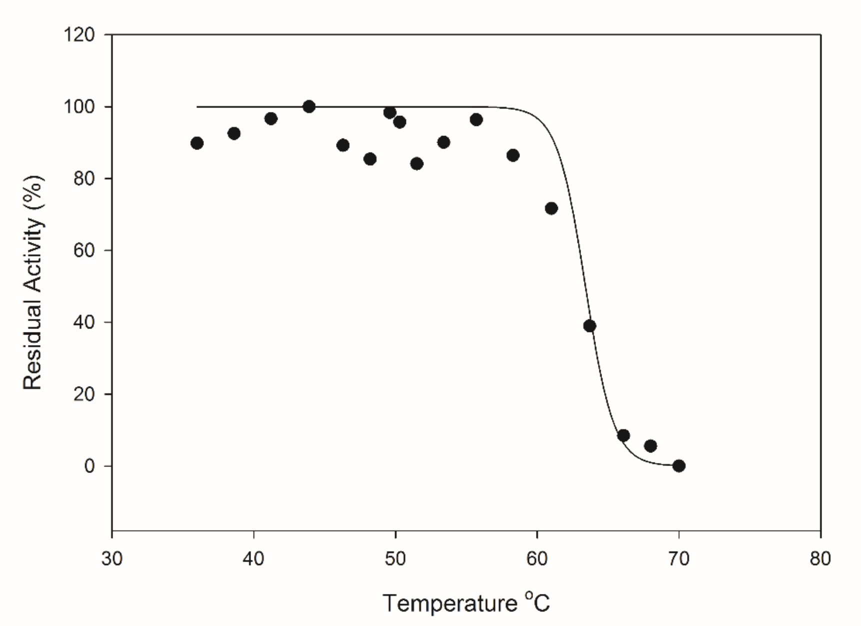

Figure S4 (b) – Triplicate replicates of a Differential Scanning Fluorimetry assay of KES23458 in 10 mM phosphate buffer. T_m is defined as the inflection point of the sigmoidal transition, and was measured to be 58.1 °C.

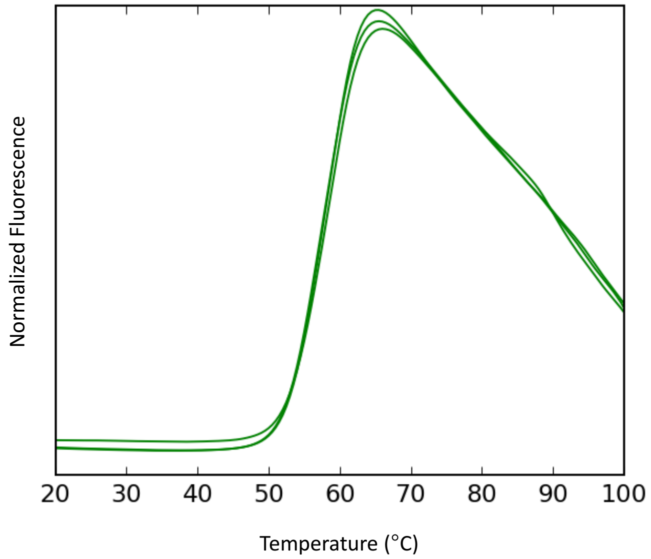

Figure S4 (c) – Triplicate replicates of a Differential Scanning Fluorimetry assay of KES23458 in a range of buffers, salts and pHs. The red dashed line is at 58 degrees (T_m in the original phosphate buffer).

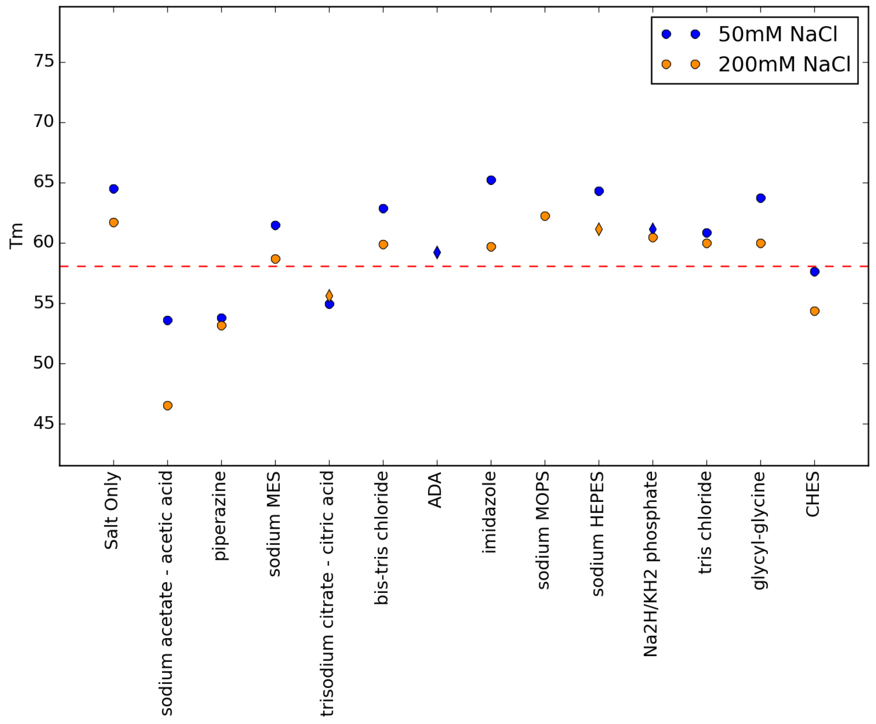

Figure S5– TA Crystal

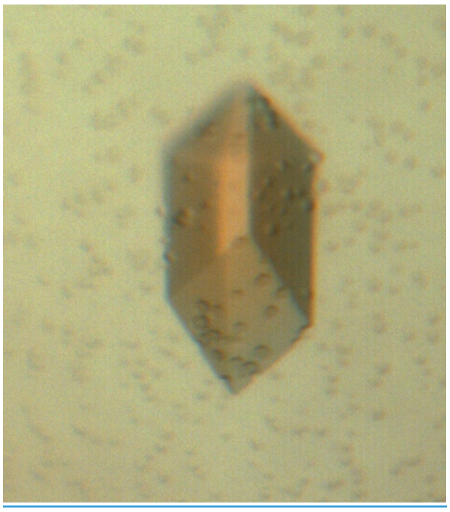

Figure S6 – Mass spectrometry data. Top: Untreated KES23458. Bottom: KES23458 treated with $NaBH_4$. m/z – 50168 – [apoprotein]$^+$. 50397 – [Holoprotein]$^+$. 50399 – [Holoprotein +2H]$^+$.

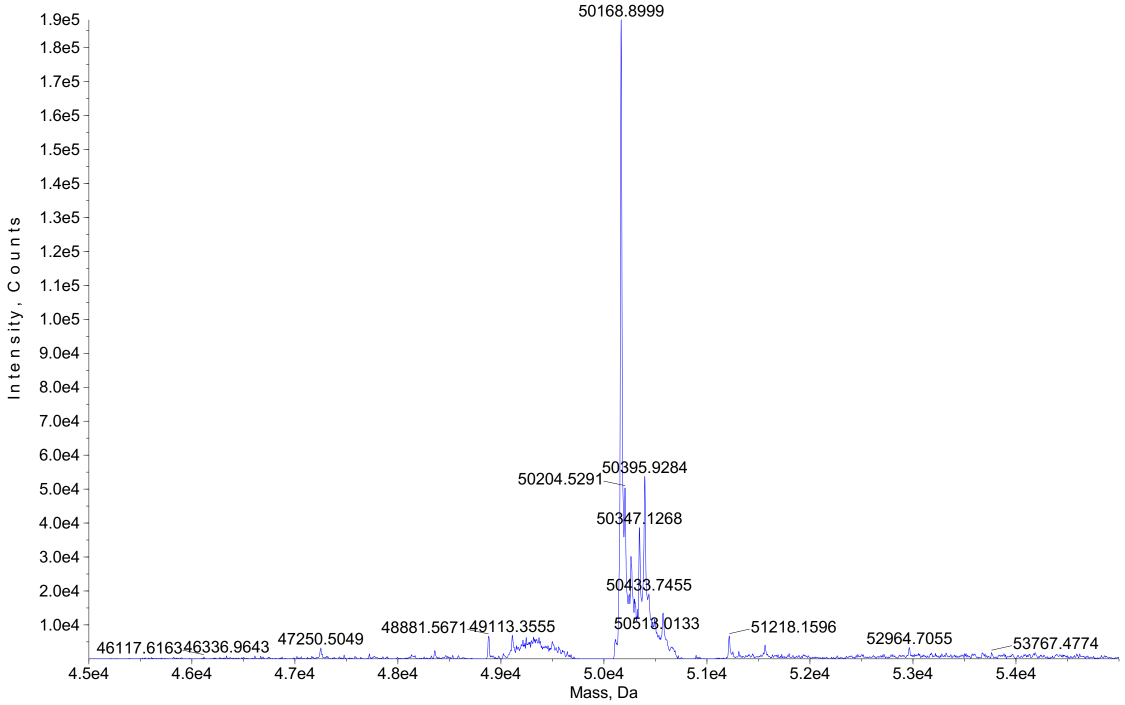

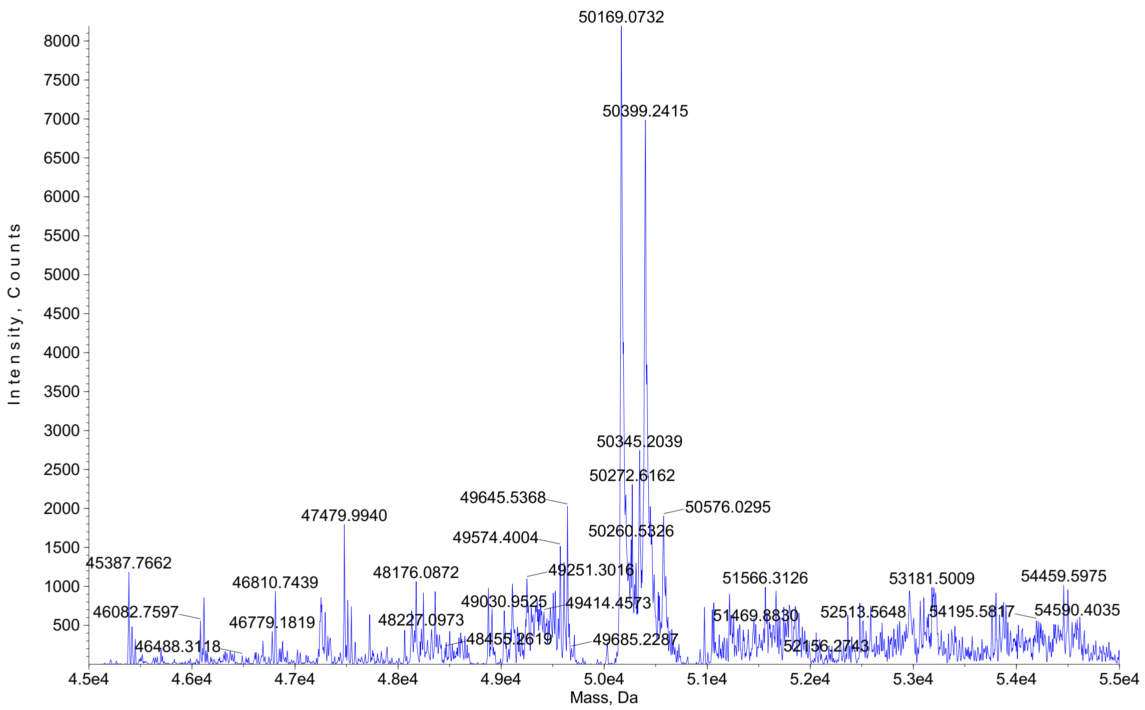

Figure S7 – RMSD analysis on KES23458 with β-alanine and 12-aminododecanoic acid docked.

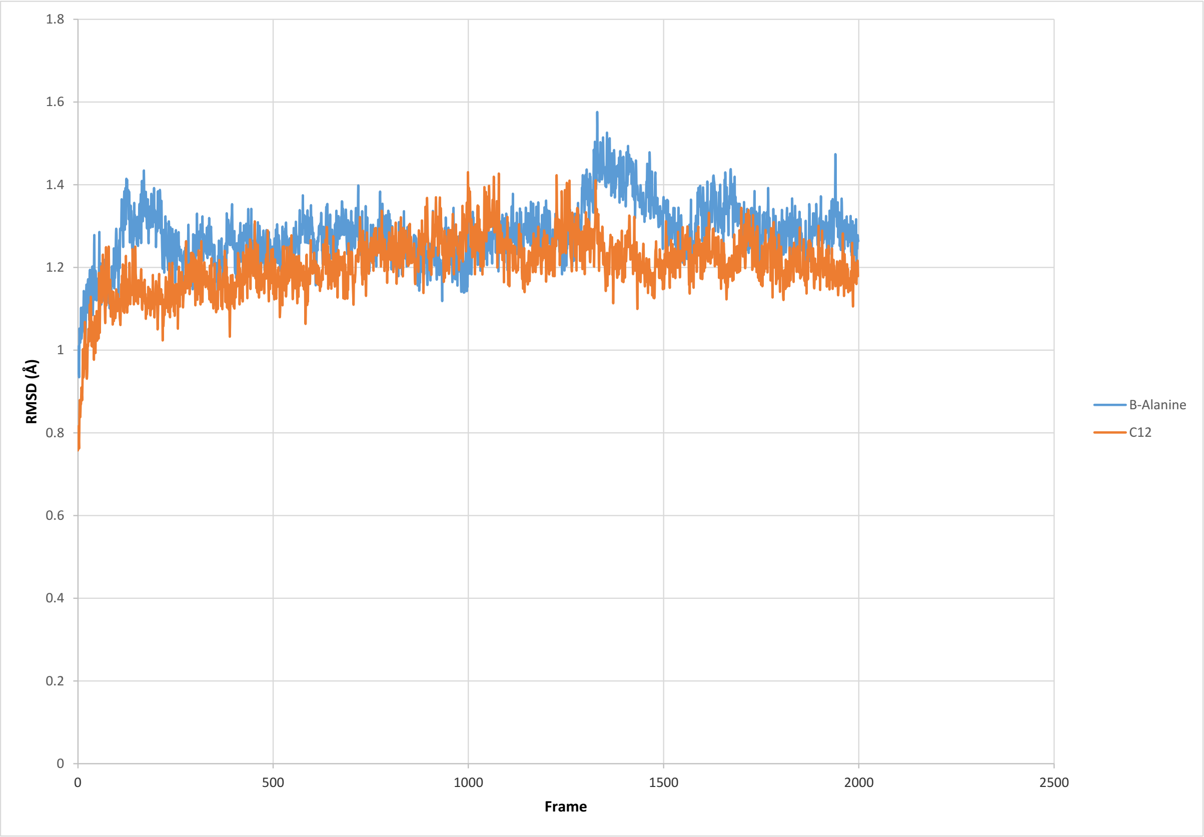

Table S1

Specific activities and relative substrate activities for KES23460.

Substrate	SA (nmol min^{-1} mg^{-1})	Relative Activity (%)
Glycine	N.D.	N.D.
B-Alanine	535.0 ± 31.3	100
D/L-Aminoisobutyric acid	15.8 ± 0.1	3.0
4-Aminobutyric acid	33.2 ± 1.0	6.1
5-Aminopentanoic acid	5.2 ± 0.3	0.7
6-Aminohexanoic acid	4.7 ± 0.6	0.6
1,3-Diaminopropane	1.4 ± 1.7	0.2
1,6-Hexamethylenediamine	4.9 ± 0.1	1.0
1,7-Heptanediamine	4.5 ± 0.4	0.8
1,8-Octanediamine	3.5 ± 0.6	0.6
1,9-Nonanediamine	4.2 ± 0.4	0.7
1,10-Decanediamine	N.D.	N.D.

Note that in each case the acceptor used was pyruvate. Assay conditions as described in Experimental section in potassium phosphate buffer (100 mM, pH 9). ND – Activity not determined

KES23458 Peptide sequence including N-terminal His_6-Tag

MGSSHHHHHHSSGLVPRGSHMNQQVNVAPSAAADLNLKAHWMPFSANRNFHKDPRIIVAAEGSWL

VDDKGRRIYDSLSGLWTCGAGHSRKEIADAVAKQIGTLDYSPGFQYGHPLSFQLAEKIAQMTPGTLD

HVFFTGSGSECADTSIKMARAYWRIKGQAQKTKLIGRARGYHGVNVAGTSLGGIGGNRKMFGPLMD

VDHLPHTLQPGMAFTKGAAETGGVELANELLKLIELHDASNIAAVIVEPMSGSAGVIVPPKGYLQRLRE

ICDANDILLIFDEVITAFGRMGKATGAEYFGVTPDIMNVAKQVTNGAVPMGAVIASSEIYDTFMNQNLP

EYAVEFGHGYTYSAHPVACAAGIAALDLLQKENLIQQSAELAPHFEKALHGLKGTKNVIDIRNCGLAGA

IQIAARDGDAIVRPFEASMKLWKEGFYVRFGGDTLQFGPTFNAKPEDLDRLFDAVGEALNGVA

KES23460 Peptide sequence including N-terminal His_6-Tag

MRGSHHHHHHGMASMTGGQQMGRDLYDDDDKGSMTLLIKHLIGGELIADTGRTADVFNPSTGEAV

RKVPLADRETMQQAIDAAKAAFPAWRNTPPAKRAQVLFRFKQLLEANEERIVKLISEEHGKTIEDAAG

ELKRGIENVEYATAAPEILKGEYSRNVGPNIDAWSDFQPIGVVAGITPFNFPAMVPLWMYPLAIACGNT

FILKPSERDPSSTLLIAELFHEAGLPKGVLNVVHGDKGAVDALIEAPEVKALSFVGSTPIAEYIYSEGTK

RGKRVQALGGAKNHAVLMPDADLDNAVSALMGAAYGSCGERCMAISVAVCVGDQIADALVQKLVPQ

IKGLKIGAGTSCGLDMGPLVTGAARDKVTGYIDTGVAQGAELVVDGRGYKVAGHENGFFLGGTLFDR

VTPEMTIYKEEIFGPVLCIVRVNSLEEAMQLINDHEYGNGTCIFTRDGEAARLFCDEIEVGMVGVNVPL

PVPVAYHSFGGWKRSLFGDLHAYGPDGVRFYTKRKAITQRWPQRKSHEAAQFAFPSNS
